# Scenario simulation of land use and land cover change in mining area

**DOI:** 10.1038/s41598-021-92299-5

**Published:** 2021-06-18

**Authors:** Xiaoyan Chang, Feng Zhang, Kanglin Cong, Xiaojun Liu

**Affiliations:** College of Information Science and Engineering, Shandong Agricultural University, Tai’an, 271018 China

**Keywords:** Ecology, Environmental sciences

## Abstract

In this study, we selected 11 townships with severe ground subsidence located in Weishan County as the study area. Based on the interpretation data of Landsat images, the Binary logistic regression model was used to explore the relationship between land use and land cover (LULC) change and the related 7 driving factors at a resolution of 60 m. Using the CLUE-S model, combined with Markov model, the simulation of LULC under three scenarios—namely, natural development scenario, ecological protection scenario and farmland protection scenario—were explored. Firstly, using LULC map in 2005 as input data, we predicted the land use spatial distribution pattern in 2016. By comparing the actual LULC map in 2016 with the simulated map in 2016, the prediction accuracy was evaluated based on the Kappa index. Then, after validation, the spatial distribution pattern of LULC in 2025 under the three scenarios was simulated. The results showed the following: (1) The driving factors had satisfactory explanatory power for LULC changes. The Kappa index was 0.82, which indicated good simulation accuracy of the CLUE-S model. (2) Under the three scenarios, the area of other agricultural land and water body showed an increasing trend; while the area of farmland, urban and rural construction land, subsided land with water accumulation, and tidal wetland showed a decreasing trend, and the area of urban and rural construction land and tidal wetland decreased the fastest. (3) Under the ecological protection scenario, the farmland decreased faster than the other two scenarios, and most of the farmland was converted to ecological land such as garden land and water body. Under the farmland protection scenario, the area of tidal wetland decreased the fastest, followed by urban and rural construction land. We anticipate that our study results will provide useful information for decision-makers and planners to take appropriate land management measures in the mining area.

## Introduction

With global environmental changes and deepening research of sustainable development, the study of Land Use and Land Cover (LULC) Change has attracted more and more attention of the researchers worldwide. The focus of research has also gradually shifted from large scale at territory or regional scale to ecologically fragile areas such as wetland^1,2^, river basin^3–6^ and mining area^7,8^.

The landscape of mining area is featured mainly by mining and other human production activities. While extensive coal mining boosts regional economy, it also brings significant environmental changes such as severe ground subsidence, land resource destruction, water resource pollution, etc., and often exacerbates the ecosystem fragility in mining area. Land rehabilitation, implying the reclamation of post-mining land subsidence, has complicated intertwined impacts, directly or indirectly, on the structure, composition and function of ecosystem in mining area. The driving force analysis can reveal the evolution law and driving mechanism of regional LULC change^5,9,10^. On this basis, scenario simulation predicts the future trend of LULC change^11–13^. Scenario simulation results not only provide theoretical foundation for the local government to formulate scientific, plausible and sustainable land use development strategies, but also have great significance for the protection of land resources and the improvement of regional ecological environment.

The research results of domestic and foreign scholars on scenario simulation models of LULC showed that single model cannot satisfy both quantitative simulation and spatial pattern analysis simultaneously. Therefore, scenario simulation is gradually changing from using single model to multiple integrated models^14–26^. Previous studies have suggested that logistic regression model can better reveal the main driving forces and interaction mechanisms of LULC change^11,26–29^. Binary logistic regression model (BLRM) is good for binary dependent variables, while multinomial logistic regression model is more suitable for multivariate dependent variables. Logistic regression model is often used for driving factors analysis of LULC change in ecologically fragile areas such as reservoir area^11,26^, mountainous area^29^, etc. And it is also mainly used for driving factors analysis of urban land use change^27,28^. Based on the results of logistic regression analysis, scenario simulation is often carried out. Using the Conversion of Land Use and its Effects at Small regional extent (CLUE-S) model, we can take into account many kinds of factors, preset multiple scenarios and visualize spatial patterns of LULC under different scenarios^30–36^.

In this study, mining area with severe ground subsidence problems was selected as the study area. Based on the 30 m interpretation data of Landsat images, firstly, 60 m was determined as the appropriate spatial resolution for driving forces analysis. At the suitable spatial resolution, BLRM was used to explore the relationship and driving mechanism between LULC types and driving factors. The results showed that the driving factors had satisfactory explanatory power for LULC changes. On this basis, using the CLUE-S model, combined with Markov model, the spatial distribution pattern of LULC in 2025 under three scenarios—namely, natural development scenario, ecological protection scenario and farmland protection scenario—was simulated and predicted. The results can guide the local government to formulate scientific and reasonable land use sustainable development strategies in land reclamation and optimal allocation of land resources, which has great significance for the rational development, utilization and protection of land resources.

The novelty of our research and scientific contributions are summarized as follows: (1) Different from the current scenario simulation research, when analyzing the relationship and driving mechanism between LULC types and driving factors, we mainly used BLRM and the entropy theory to determine the appropriate spatial resolution of 60 m. However, most of the related researches only determined the research scale subjectively, which was lack of scientificity and basis. (2) Before the scenario simulation of LULC in the future, firstly, using LULC map in 2005 as input data, we predicted the spatial distribution pattern of LULC in 2016. By comparing the actual LULC map in 2016 with the simulated map in 2016, the prediction accuracy was evaluated. On the basis of the prediction results meeting the accuracy requirements, natural development scenario, ecological protection scenario and farmland protection scenario were designed, and we simulated and predicted the futural LULC spatial distribution pattern of the mining area, which increased the accuracy and credibility of the prediction results. Our study process also provides a reference for the related research of scenario simulation.

**Figure 1:**
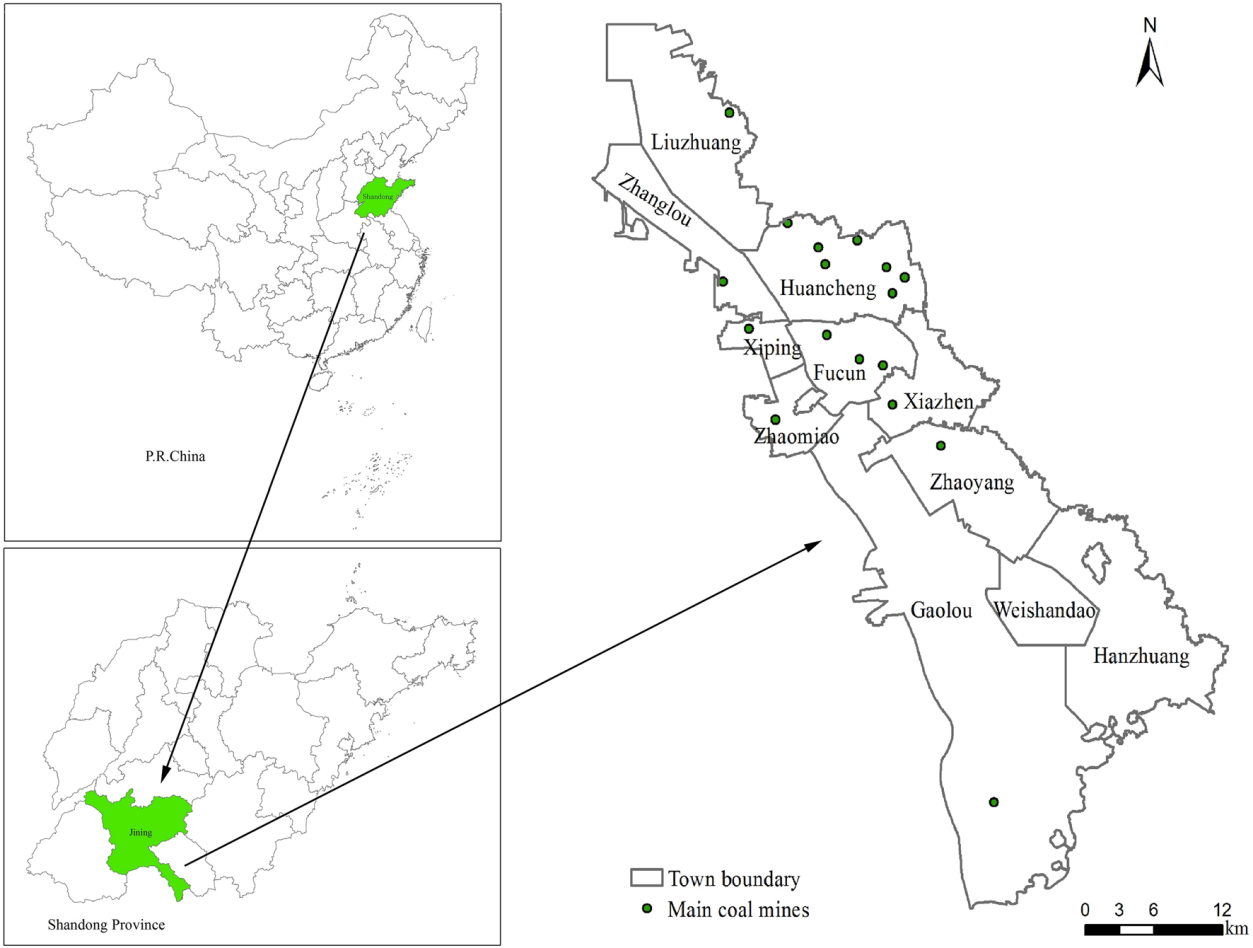
Geographical location and mining area distribution in the research area. Maps were generated using ArcGIS 10.1 for Desktop (http://www.esri.com/software/arcgis/arcgis-for-desktop).

## Study area

Weishan county($34^\circ 27$′N to $35^\circ 20$′N, $116^\circ 34$′E to $117^\circ 24$′E), is located in the southern part of Jining City, Shandong Province. The study area is 120 km long from north to south, 8–30 km wide from west to east. Nansi Lake, the largest freshwater lake in northern China, is located within the study area. Weishan county comprises 3 sub-districts, 10 towns, 2 townships and 1 economic development zone (2014 administrative division), with a total area of 1779.8 km^2^. We selected 11 townships with a total area of 1176.86 km^2^, because this study area has more mines, more severe land subsidence, and is spatially coherent. The geographical location of the study area and the distribution of mining area are shown in Fig. 1.

## Data and methods

### Data source and preprocessing

Considering factors such as amount of cloud and time intervals of image, four remote sensing images with a spatial resolution of 30 m, including Landsat 5 Thematic Mapper (TM) images for 08-21-2000, 09-04-2005 and 09-18-2010, and Landsat 8 Operational Land Imager (OLI) for 09-02-2016,were obtained from the Geospatial Data Cloud Platform (http://www.gscloud.cn). LULC information was extracted from these remote sensing images. In addition, the digital elevation model (DEM) with a spatial resolution of 30 m was obtained from the website. Elevation and slope information were derived from DEM data and used as terrain driving factors for scenario simulation. Other supporting data, such as Weishan County land use data, mine distribution data, general land use planing (2006–2020) and mineral resources planning (2008–2015), Jining City coal mining subsidence land rearrangement planning (2016–2030), were obtained from Weishan Natural Resources and Planning Bureau. These data were used for better data analysis.

Considering severe ground subsidence and seeper in the study area, and referring to national standards: *Current Land Use Classification* (GB/T 21010-2017), remote sensing images were interpreted into six LULC types: farmland, other agricultural land, urban and rural construction land, subsided seeper area, water area, and tidal wetland.

In the process of image interpretation, firstly, the remote sensing image was divided into two regions: one region were the lake and the surrounding tidal wetland, and the other region included farmland, other agricultural land, urban and rural construction land, subsided seeper area, etc.

In region 1, decision tree classification, combined with the Modified Normalized Difference Water Index (MNDWI), was used to extract lakes. Then we masked them in region 1. The Normalized Difference Vegetation Index (NDVI) was calculated for the remaining image of region 1. Tidal wetland was mainly distributed along rivers and lakes, and NDVI value was higher than that of farmland and other vegetation. By analyzing its geographical distribution and NDVI value, and referring to Weishan County land use data, the appropriate threshold was selected to extract tidal wetland.

The spectral signature of rivers, ditches and aquaculture ponds in other agricultural land in region 2 could be easily distinguished from other surface features. They could be extracted step by step by manual visual interpretation and empirical knowledge, referring to Weishan County land use data and water system data. Then we masked them separately in region 2. After extracting rivers, ditches, aquaculture ponds with high water content, the remaining LULC type with high water content in region 2 was subsided seeper area. According to the relationship of spectral signature of different LULC types, it was concluded that among the remaining LULC types in region 2, only TM3 band value of subsided seeper area was higher than TM5 band value. Using this characteristic, subsided seeper area could be distinguished from other LULC types. After extracting subsided seeper area, the remaining LULC types in region 2 were farmland and urban and rural construction land. The spectral characteristics of them were very different. Therefore, they could be distinguished using support vector machine (SVM) classification method, and their respective binary images were generated using decision tree method.

**Figure 2:**
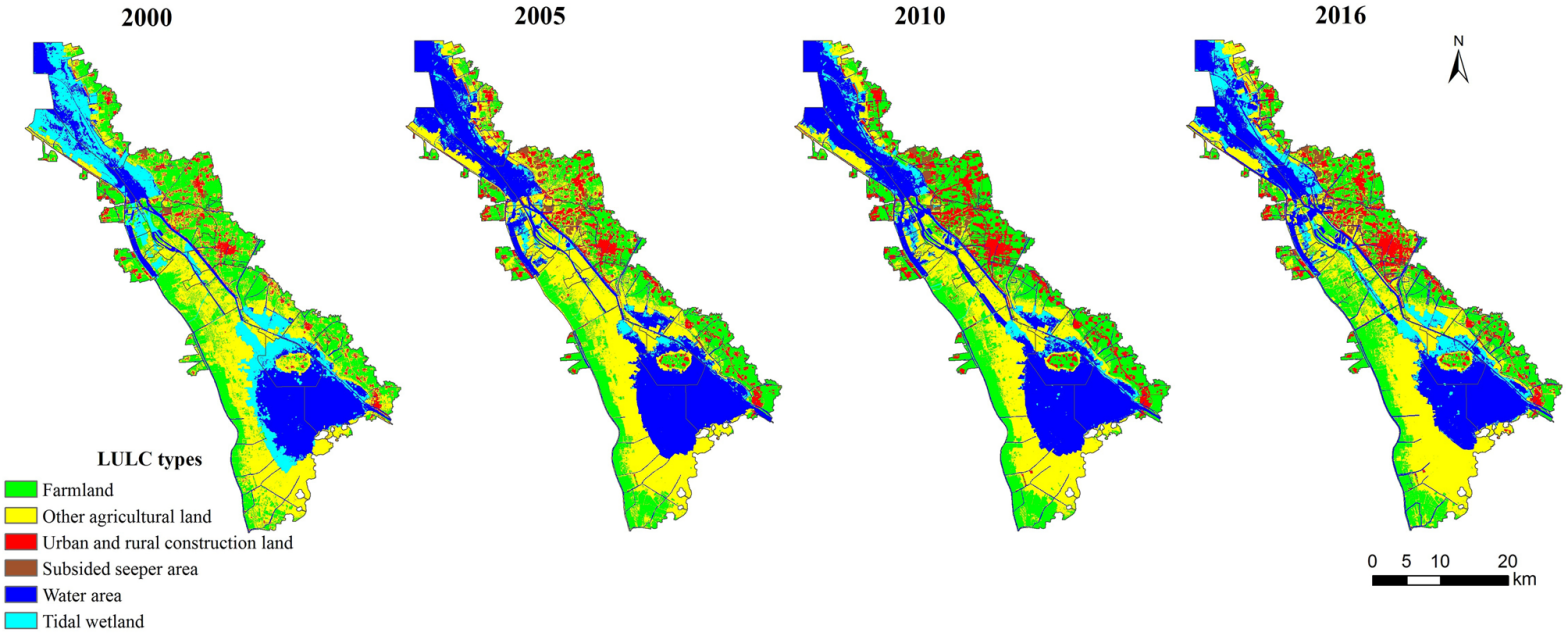
The LULC types maps of 2000, 2005, 2010 and 2016. Maps were generated using ArcGIS 10.1 for Desktop (http://www.esri.com/software/arcgis/arcgis-for-desktop).

The extracted six LULC types were shown in single layer and binary form respectively. Six LULC types were coded and synthesized into one image. We obtained 2000, 2005, 2010, 2016 LULC type maps (Fig. 2). Finally classification post-processing and accuracy evaluation were operated.

The accuracy of the interpretation results was verified by confusion matrix and kappa coefficient. The kappa coefficients of the four interpretation maps were 0.84, 0.85, 0.82 and 0.86, respectively (Table 1). The accuracy could meet the needs of further research.

**Table 1:** Accuracy evaluation of the interpretation results (%).

LULC types	2000		2005		2010		2016	
	Producer accuracy	User accuracy	Producer accuracy	User accuracy	Producer accuracy	User accuracy	Producer accuracy	User accuracy
Farmland	87.31	85.15	89.91	83.62	84.32	83.31	91.17	89.20
Other agricultural land	84.51	85.89	81.57	80.45	80.00	82.60	83.33	85.71
Urban and rural construction land	89.74	88.83	93.01	92.89	90.50	89.89	93.75	92.66
Subsided seeper area	84.58	88.54	84.92	88.43	86.97	87.75	88.49	86.28
Water area	90.82	92.04	88.63	89.60	85.23	89.44	86.14	90.31
Tidal wetland	83.53	81.38	80.66	84.65	82.59	79.43	81.57	85.51
Overall Accuracy	86.91		89.16		87.12		89.51	
Kappa Coefficient	0.84		0.85		0.82		0.86	

By reading previous research results^37–41^, based on the entropy theory, in the same study area, high spatial resolution data contains more entropy than low spatial resolution data, and reflecting more detailed information, but it will increase the uncertainty of prediction results and reduce the prediction accuracy. Although the prediction accuracy of low spatial resolution data increases, it will lose lots of detailed information. In order to ensure the accuracy of the simulation, considering the area of the study area and data requirement of the CLUE-S model, the interpreted LULC maps with a resolution of 30 m exceed the upper limit of the CLUE-S model data requirement, so the LULC maps were resampled to multiple scales including 60 m, 90 m, 120 m, and 150 m to facilitate logistic regression analysis of LULC types and driving factors.

### Selection and processing of driving factors

To interpret the relationship between the LULC and its driving factors in the mining area, we not only need to identify the driving factors that have greater explanatory power for LULC change, but also need to quantitatively describe the relationship between driving factors and LULC types.

Considering the accessibility, usability of the data and the actual conditions in the study area, seven driving factors were selected based on the land use map of Weishan County in 2005 and the DEM data^5,10,11,13,26,28–30^. The driving factors included: (1) terrain factors, including elevation and slope factors; (2) five accessibility factors, including the nearest distance between each grid pixel and the main roads, the major rivers, the residential area, the major mines, and the ditches. The 30 m grid data of each driving factor were resampled to 60 m, 90 m, 120 m and 150 m respectively.

In this study, BLRM was used to explore the relationship between LULC change and the related 7 driving factors. BLRM is sensitive to multicollinearity. In order to eliminate the influence of collinearity on the regression results, the multicollinearity between independent variables was diagnosed before the regression model was established.

The receiver operating characteristic (ROC) curve was used to evaluate the accuracy of regression analysis results at different scales. The results showed that using 60 m resolution provided more accurate regression analysis results and suffered less loss of LULC and driving factor information during resampling. Therefore, we used 60 m × 60 m grid cell data to driving forces analysis.

**Figure 3:**
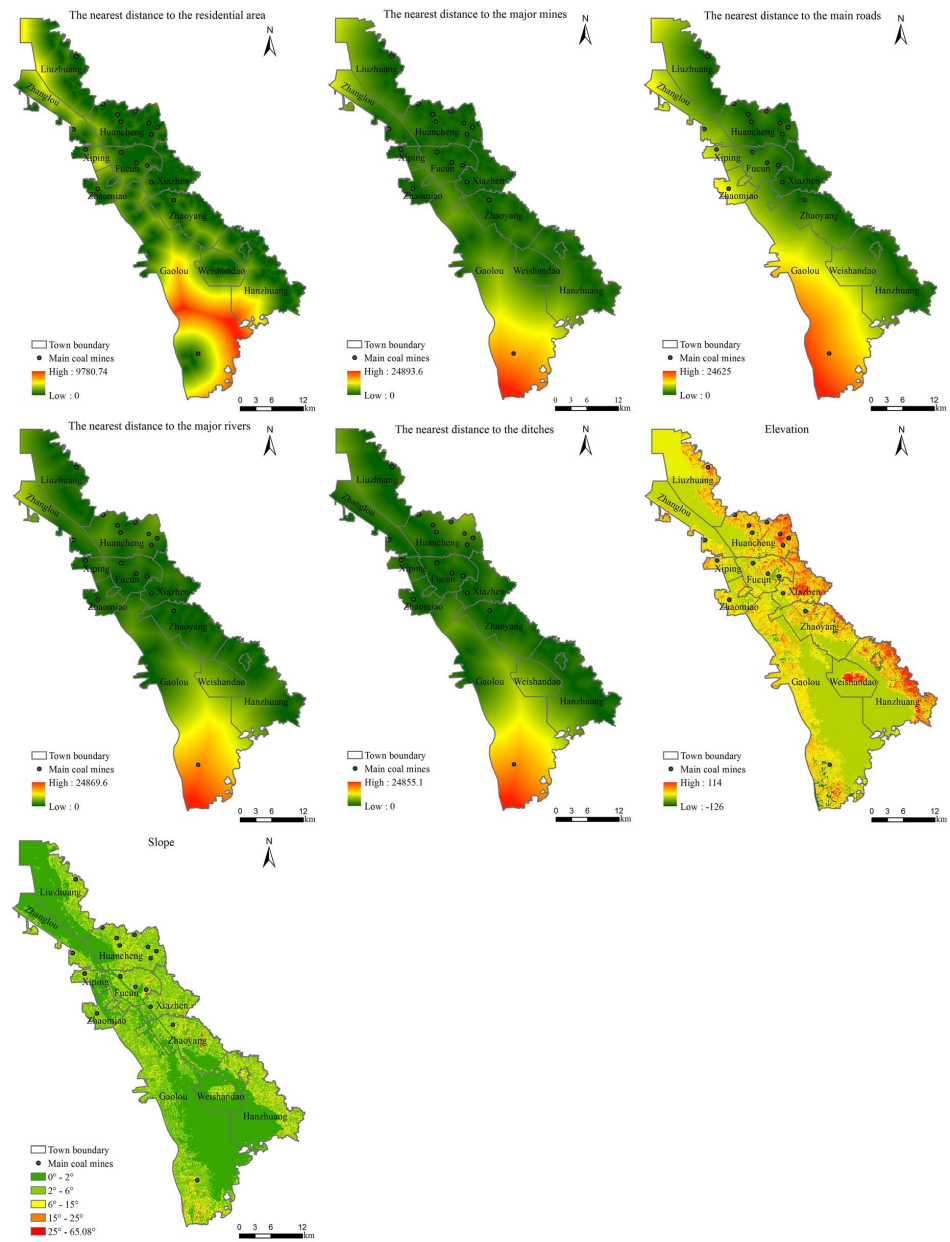
Raster maps of driving factors at a resolution scale of 60 m. Maps were generated using ArcGIS 10.1 for Desktop (http://www.esri.com/software/arcgis/arcgis-for-desktop).

Raster maps of each driving factor at a resolution scale of 60 m are shown in Fig. 3.

### Logistic regression analysis of LULC types and driving factors

BLRM is often used for regression analysis of explanatory binary variables. The presence and absence of a certain type of LULC in a specific area is set as 1 and 0, respectively, which is characteristic for binary variable. Therefore, we used BLRM to calculate the probability (*P*) of various LULC types in a specific spatial location, and its mathematical expression is:

1 $$\begin{aligned} \ln \left( \frac{P}{1-P}\right) = \beta _0 + \beta _1 X \end{aligned}$$

where $\frac{P}{1-P}$ is the ’odds ratio’ of an event, abbreviated as $\Omega$, which represents the odds that an outcome will occur given a particular condition compared to the odds of the outcome occurring in the absence of that condition; $\beta _0$ is a constant; $\beta _1$ is the correlation coefficient of an explaining variable and an explained variable. Making mathematical transformation of the above expression, we get: $\Omega = (\frac{P}{1-P}) = e^{\beta _0 + \beta _1 X}$.

Regression analysis using BLRM, we divided the study area into many grid cells. Taking each LULC type as the explained variable, and the driving factor causing LULC change as the explanatory variable, we calculated the odds ratio of each LULC type in a specific spatial location, and analyzed the relationship between each LULC type and the driving factors. The calculating equation is:

2 $$\begin{aligned} \mathrm{Logit} P = \ln \left( \frac{P_i}{1-P_i}\right) = \beta _0 + \beta _1 X_{1,i} + \beta _2 X_{2,i} + \cdots + \beta _n X_{n,i} \end{aligned}$$

Making mathematical transformation of the above equation, we get:

3 $$\begin{aligned} P_i = \frac{e^{(\beta _0 + \beta _1 X_{1,i} + \beta _2 X_{2,i} + \cdots + \beta _n X_{n,i})}}{1+e^{(\beta _0 + \beta _1 X_{1,i} + \beta _2 X_{2,i} + \cdots + \beta _n X_{n,i})}} \end{aligned}$$

where: $P_i$ is the probability of a certain LULC type *i* in a grid cell, $X_{1,i}\sim X_{n,i}$ are the driving factors of LULC type *i*, $\beta _0$ is the constant, $\beta _1\sim \beta _n$ are the correlation coefficients of each driving factor and LULC type *i*.

The receiver operating characteristic (ROC) was used to evaluate the accuracy of regression analysis results. The accuracy can be measured by calculating the area under the ROC curve. The area value is between 0.5 and 1. The closer the value is to 1, the higher the accuracy is. In general, the area under the ROC curve is greater than 0.7, which indicates that the selected factor has good explanatory power^27,42^.

### CLUE-S simulation and accuracy validation

Before using the CLUE-S model for futural LULC scenario simulation in mining area, the prediction accuracy needs to be verified. Based on the data of LULC in 2005, the spatial distribution pattern of LULC in 2016 was predicted firstly.

The modeling accuracy was evaluated based on the Kappa index by comparing the actual LULC map in 2016 with the simulated in 2016^27,43,44^. Equation (4) gives one of the most popular Kappa index equations: i.e.,

4 $$\begin{aligned} \mathrm{Kappa}=\frac{P_o-P_c}{P_p-P_c} \end{aligned}$$

where $P_o$ is the observed proportion correct, $P_c$ is the expected proportion correct due to chance, $P_c$ =1/*n*, *n* is the number of LULC types, and $P_p$ is the proportion correct when classification is perfect.

In order to further verify the accuracy of the model simulation, we also calculated kappa for quantity (Kquantity).

### Scenario setting of futural LULC simulation

Due to the continuous population growth and mineral exploitation in the study area, the land resources, especially farmland resources, have become increasingly scarce and the environment has been deteriorating. Based on the simulation and validated results during 2005-2016, we defined three scenarios—namely natural development scenario, ecological protection scenario, and farmland protection scenario—to predict LULC spatial patterns for 2025.

#### Natural development scenario

In this scenario, the land use demand of the study area was basically not restricted by policies in near future. We assumed that the change rate of each LULC type in near future was consistent with the change trend from 2005 to 2016. So it is defined as natural development scenario. Using Markov model to obtain the area transition probability matrix of each year from 2017 to 2025, and taking the proportion of each LULC type area in the total study area in 2005 as the initial state matrix, the area of each LULC type in 2025 under the natural development scenario was predicted.

Based on the characteristics and trend of the LULC change from 2005 to 2016, after appropriately adjusting the transition probability matrix of different LULC types, we predicted the demands of each LULC type in 2025 under ecological protection scenario and farmland protection scenario using Markov model^45,46^.

#### Ecological protection scenario

This scenario emphasizes protecting the ecological environment, restricting the conversion of the LULC types that have more regulatory effects on the ecosystem, such as tidal wetland and water area, to other land use types. Garden land, woodland, grassland, and aquaculture land, belong to other agricultural land, which have regulatory effects on the local ecosystem, so their conversion to other LULC types should be restricted as well.

#### Farmland protection scenario

According to the guidelines of “the general land use planning in Weishan County (2006-2020)”, we should maximize the potential use of current construction land, implement intensive and economical utilization of construction land, and use less or not use farmland to economical construction. So in order to ensure the dynamic balance of total farmland amount and the regional food supply security, in the farmland protection scenario, the conversion from farmland to other land use types should be restricted. The projected land use demands for 2025 under the three different scenarios are shown in Table 2.

**Table 2:** Areas of LULC types in 2025 under different scenarios (ha).

Scenarios	LULC types					
	Farmland	Other agricultural land	Urban and rural construction land	Subsided seeper area	Water area	Tidal wetland
Natural development scenario	25907.39	41817.72	6642.7	1119.64	39199.37	2995.01
Ecological protection scenario	24535.92	42839.93	6385.32	1020.68	39362.02	3537.96
Farmland protection scenario	28074.34	40163.25	6449.67	1099.93	38918.11	2976.55

## Results

### Multicollinearity diagnostic result of driving factors

In this study, tolerance and variance inflation factor were used to diagnose the multicollinearity of driving factors. The results are shown in Table 3.

**Table 3:** Multicollinearity diagnosis of driving factors.

Driving factors	Tolerance	Variance inflation factor
The distance to residential area	0.563	1.777
The distance to mines	0.351	2.846
The distance to roads	0.228	4.387
The distance to rivers	0.207	4.833
The distance to ditches	0.329	3.037
Elevation	0.888	1.126
Slope	0.874	1.144

The minimum tolerance of the seven driving factors was 0.207, which was greater than the critical value of 0.1. The maximum variance inflation factor was 4.833, less than the critical value of 5. It showed that there was no multicollinearity relationship among the seven driving factors.

### Regression analysis result of LULC types and driving factors

The relationship between each LULC type and the driving factors was obtained using BLRM^11,29^. Firstly, the zero-mean normalization method was used to standardize the driving factors data. The *β* coefficients (listed in Table 4), derived from the logistic regression equation, were used as input parameters for the CLUE-S model. Table 4 shows that the distance to residential area was the main driving factor for the change of urban and rural construction land, and there was obvious negative correlation between them, which suggested the probability of construction land occurrence was relatively less in areas far away from the residential area. There was a significant negative correlation between subsided seeper area and the distance from mines, main rivers, and roads, suggesting that the probability of subsidence water area occurrence increased around mines, rivers and main roads. The distance to river was a negative explanatory variable for other agricultural land, suggesting that areas far away from major rivers would show smaller probability of other agricultural land. In particular, aquaculture land is one of the land use types of other agricultural land, aquaculture land area would drop significantly as the distance to river increased. The distances to major ditches and roads were significant negative explanatory variables for water area and tidal wetland.

**Table 4:** Regression coefficient (β) of each LULC type and driving factors.

Explanatory variable	Farmland	Other agricultural land	Urban and rural construction land	Subsided seeper area	Water area	Tidal wetland
The distance to residential area	− 0.974	0.136	− 3.282	1.197	1.564	0.562
The distance to mines	-	0.316	− 1.112	− 5.813	− 0.419	1.274
The distance to roads	0.894	− 0.101	− 0.216	− 0.833	− 1.221	− 0.603
The distance to rivers	− 1.769	− 0.738	-	− 1.562	3.396	−
The distance to ditches	1.588	0.655	0.207	0.481	− 3.337	− 2.287
Elevation	− 0.19	0.045	0.044	0.571	0.214	0.225
Slope	− 0.136	0.104	− 0.057	−	0.051	0.275
Constant	− 0.009	− 1.284	− 5.483	− 8.94	− 1.779	− 4.237

**Figure 4:**
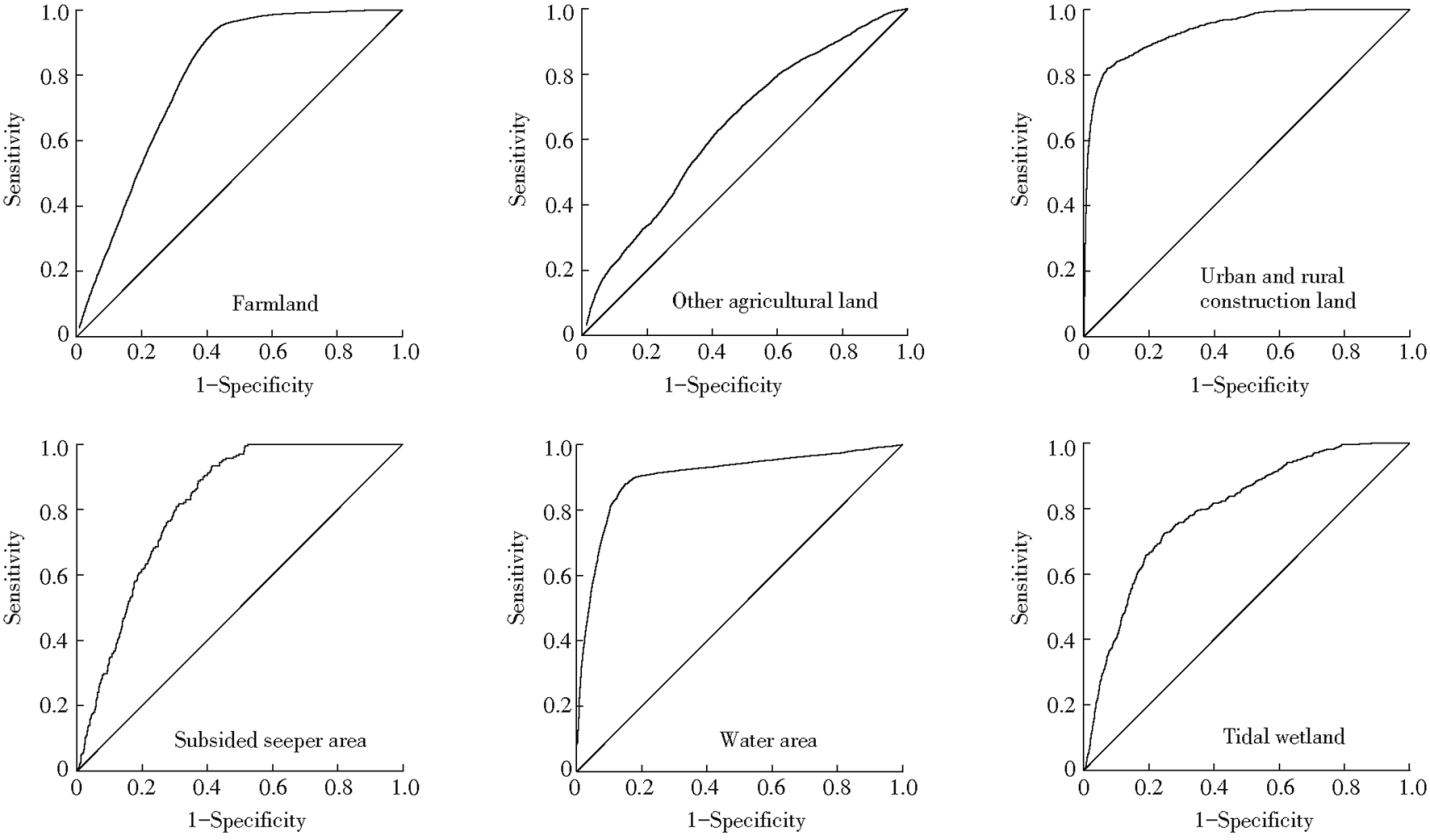
ROC curves for regression analysis of LULC type and driver factors.

The area values under the ROC curve were as shown in Fig. 4: farmland 0.793, other agricultural land 0.639, urban and rural construction land 0.940, subsidence seeper area 0.815, water area 0.903, tidal wetland 0.795. Except for other agricultural land, the values of ROC of other LULC types were above 0.70, which suggested the selected driving factors could better simulate the spatial pattern of land use. The probability distribution of the simulated land use types was consistent with that of the actual land use types. The ROC value of other agricultural land was slightly lower, the reason may be that other agricultural land includes garden land, woodland and grassland, so the simulation effect was not good.

### Accuracy validation of scenario simulation results in 2016

The spatial overlay analysis of the simulated LULC map and the real map in 2016 was carried out, and the calculated $P_o$ value was 0.857. In this study, the land use types were 6, so $P_c$ =1/6. $P_p$ is the correct simulation proportion under the ideal classification, $P_p$ =1. So the Kappa index was calculated as 0.829, larger than 0.75. Kquantity was 0.978. Those indicated satisfactory accuracy and suggested that the CLUE-S model could be used to simulate the LULC change in the future under different scenarios.

### Prediction of futural LULC spatial distribution pattern under different scenarios

The demands of each LULC type under three different scenarios were input into the CLUE-S model. Meanwhile, according to the LULC change in the study area from 2000 to 2016, the conversion elasticity values of each LULC type in the future scenario simulation were preliminarily determined. During the simulation, by comparing the simulation results with the set scenarios, the conversion elasticity values were repeatedly adjusted. Finally, the conversion elasticity coefficient values of each LULC type under three different scenarios were determined as shown in Table 5.

**Table 5:** The ELAS of LULC types under different scenarios.

LULC types	Natural development scenario	Ecological protection scenario	Farmland protection scenario
Farmland	0.7	0.7	0.8
Other agricultural land	0.5	0.6	0.5
Urban and rural construction land	0.9	0.9	0.9
Subsided seeper area	0.6	0.5	0.6
Water area	0.6	0.7	0.6
Tidal wetland	0.55	0.6	0.5

**Figure 5:**
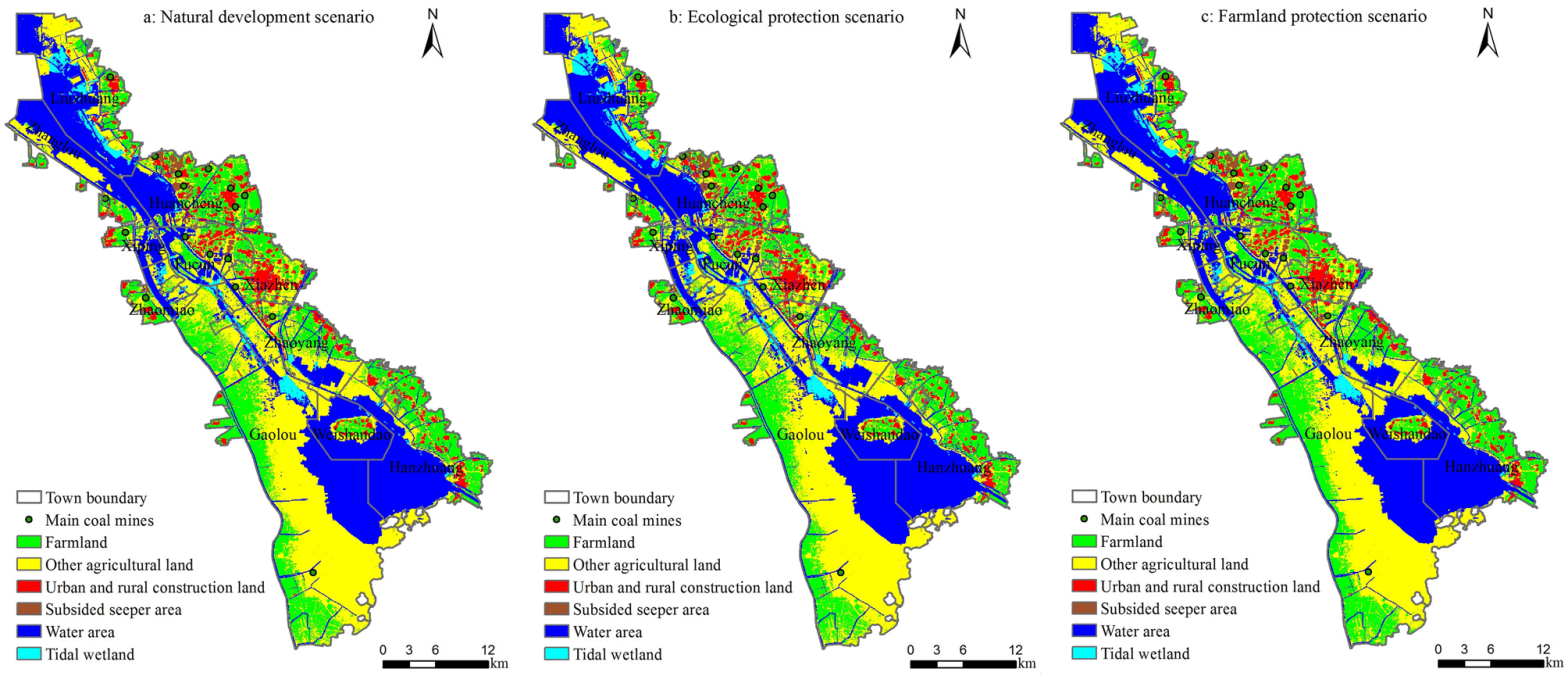
LULC simulation maps in 2025 under different scenarios. Maps were generated using ArcGIS 10.1 for Desktop (http://www.esri.com/software/arcgis/arcgis-for-desktop).

The BLRM was established and validated to explore the relationship between driving factors and LULC types. Using the selected 7 driving factors and LULC data in 2016 as input data for simulation, the spatial distribution of each LULC type in 2025 under three different scenarios were predicted after fine-tuning configuration of model parameters. The prediction results are shown in Table 6 and Fig. 5.

**Table 6:** Dynamic change of LULC types under different scenarios from 2017 to 2025.

LULC types	Natural development scenario		Ecological protectio scenario		Farmland protection scenario	
	Area change (ha)	Single dynamics (%)	Area change (ha)	Single dynamics (%)	Area change (ha)	Single dynamics (%)
Farmland	− 2801.25	− 1.95	− 4172.49	− 2.91	− 629.73	− 0.44
Other agricultural land	5515.83	3.04	6548.31	3.61	3867.03	2.13
Urban and rural construction land	− 3168.18	− 6.46	− 3434.58	− 7.01	− 3370.5	− 6.88
Subsided seeper area	− 65.61	− 1.12	− 166.05	− 2.84	− 85.05	− 1.46
Water area	7817.13	4.98	7978.77	5.08	7528.41	4.79
Tidal wetland	− 7302.15	− 14.2	− 6758.19	− 13.14	− 7314.39	− 14.23

### LULC characteristics in the future

As shown in Table 6 and Fig. 5, other agricultural land and water area increased under the three scenarios. It showed that ecological land, such as other agricultural land and water area, which play an important role in regulating the regional ecological environment, has attracted more and more attention from 2017 to 2025. Farmland, urban and rural construction land, subsided seeper area and tidal wetland showed a shrinking trend. And the single dynamic degree of tidal wetland and urban and rural construction land were the largest, − 14.23% and − 7.01% respectively. There are several possible explanations for the observed quick shrinkage of urban and rural construction land and tidal wetland. First of all, with the gradually increased utilization of tidal wetland and other unused land, some tidal wetland could be developed into aquaculture land or artificial wetland, which is also consistent with the current change trend of tidal wetland. Secondly, under the proposing of intensive and economical utilization of construction land, some abandoned industrial and mining land could be gradually reclaimed into usable garden land, forest land and other agricultural land. The single dynamic degree of farmland was the greatest in the ecological protection scenario. The result indicated that under this scenario, farmland decreased faster than the other two scenarios. This accelerated reduction of farmland area was probably due to the implementation of “Grain for Green Project” and “Grain for water Project”, and other ecological environmental protection measures. During 2017–2025, the area of projected subsided seeper also gradually reduced because of the advancement of land reclamation and the improvement of technology.

**Figure 6:**
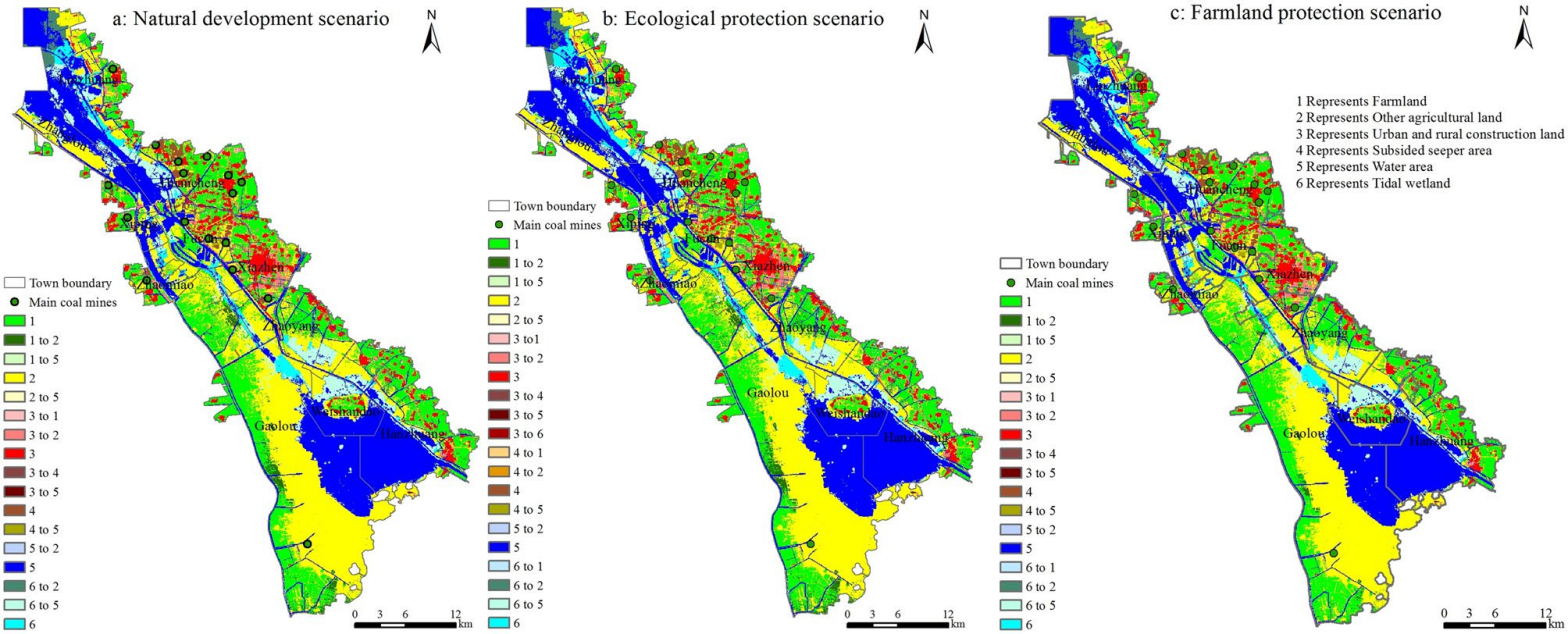
LULC changes under different scenarios from 2017 to 2025. Maps were generated using ArcGIS 10.1 for Desktop (http://www.esri.com/software/arcgis/arcgis-for-desktop).

To further analyze the LULC change in 2025, the simulated LULC maps in three different scenarios and land use map in 2016 were subjected to raster calculation. The results are shown in Fig. 6.

#### Natural development scenario

As shown in Fig. 6 and Table 6, under the natural development scenario, some farmland concentrated in the east of Wanglou Village of Gaolou Township and surrounding areas, was projected to be converted to garden land or other agricultural land in 2025, due to its natural geological characteristics or adjustment of agricultural structure. Other farmland, located in the tributaries of Weishan Lake and surrounding areas southern to the secondary dam, was projected to be converted to water body, due to rainfall and resulting lake water level rise. The area of construction land in the south of Xiazhen Street and the north of Zhaoyang Street was projected to decrease, and it was mainly transferred into other agricultural land. Fucun Street has severe land subsidence and is close to lake. After reclamation, some of the subsided land with water accumulation was projected to be converted to water area. Tidal wetland was mostly predicted to be converted to other agricultural land or water area. Specifically, the large areas of tidal wetland, located in the east bank of Zhaoyang Lake and the north bank of Weishan Lake, were projected to be converted to water area, and a large area of tidal wetland in the north of Liuzhuang Town was projected to be converted to other agricultural land.

#### Ecological protection scenario

In the ecological protection scenario, the change of LULC types was similar to those in the natural development scenario. Urban and rural construction land and tidal wetland decreased the fastest. A large area of construction land in the east of Xiazhen Street and the middle of Zhaomiao Township was projected to be converted to other land. In this scenario, the reduction of construction land was faster than that in the other two scenarios, with -7.01% changing rate and total area reduction of 3434.58 ha. The tidal wetland was mostly to be converted to water body. In addition, the reduction of farmland was also faster in this scenario as compared with the other two scenarios, with an estimated changing rate of − 2.91% and a total area reduction of 4172.49 ha. The farmland was mainly converted to more ecological land types such as garden land and water area, due to the implementation of “Grain for Green Project ” and “Grain for water Project”. The subsided land with water accumulation also had a faster conversion rate in this scenario and was mostly to be converted to water area.

#### Farmland protection scenario

In this scenario, the reduction rate of farmland dropped significantly, with a small changing rate of -0.44% and a total area reduction of 629.73 ha. And in the northeast of Huancheng Town, some of the construction land was projected to be converted to farmland, which contributed to the farmland preservation. Both urban and rural construction land and tidal wetland showed deceasing trends, which are similar to those in the natural development scenario and the ecological protection scenario. However, the tidal wetland was projected to have the fastest changing rate in this scenario. A small proportion of tidal wetland located in the northern part of Huancheng Town was projected to be converted to farmland. Due to the effective farmland preservation measures, the changes of garden land and other agricultural land were significantly less in this scenario as compared with the other two scenarios. The change of water area in this scenario was similar to that and slightly slower than that in the natural development scenario, but significantly different from that in the ecological protection scenario. The subsided land with water accumulation changed in a similar decreasing trend in the three scenarios.

## Discussion and conclusion

### Discussion

In this study, the application of the CLUE-S model, combined with Markov model and BLRM, suggests that this method can reveal the driving factors of LULC change at a resolution of 60 m, and can effectively simulate the multi scenario of land use in the future. The results can guide the government to make more reasonable allocation of land resources in the mining area. In near future, in order to ensure the regional food supply security, Weishan’s government should enforce the management of farmland resources, especially high quality cultivated field, control the increase of construction land and implement the intensive and economical use of construction land. Meanwhile, the ecological LULC types, such as other agricultural land, water body and tidal wetland, should maintain a balanced proportion in the mining area. And the subsided land with water accumulation should be effectively reclaimed using appropriate technologies, in order to ensure the sustainable utilization of land resources and improve the ecological environment in the mining area.

However, due to the limitation of data acquisition, we should need to further improve the comprehensiveness of driving factors. Therefore, we should need to incorporate policies, measures, as well as other human factors in future research to better analyze the driving forces of land use dynamic changes. Markov model was used to predict the land use demand in the future in this study, we did not account for both random and systematic LULC transitions^47^. This is also what we need to further improve in the future study of LULC change.

### Conclusion

In this study, using the CLUE-S model, combined with Markov model and BLRM, the spatial distribution pattern of LULC in 2025 under different scenarios was simulated and predicted. The characteristics of LULC change in 2025 are as follows:

(1) Under the three scenarios, the area of other agricultural land and water body which have regulatory effect on regional ecosystem showed an increasing trend; while the area of farmland, urban and rural construction land, subsided seeper area, and tidal wetland showed a decreasing trend, and the area of urban and rural construction land and tidal wetland decreased the fastest from 2017 to 2025. Under the ecological protection scenario, the decrease of farmland was faster than that in the other two scenarios. The projected area of subsided land with water accumulation also reduced gradually because of the advancement of reclamation.

(2) Under the natural development scenario, some farmland concentrated in the east of Wanglou Village of Gaolou Township and surrounding areas, was projected to be converted to garden land or other agricultural land. Some farmland, located in the tributaries of Weishan Lake and surrounding areas southern to the secondary dam, was projected to be converted to water body. Fucun Street has severe land subsidence and is close to lake. After reclamation, some of the subsided land with water accumulation converted to water body. Tidal wetland was mostly converted to other agricultural land or water body. The construction land was mainly converted to other agricultural land.

(3) Under the ecological protection scenario, the changes of LULC types were similar to the natural development scenario, but the change speed was faster than the other two scenarios. Among all LULC types, urban and rural construction land decreased the fastest. Farmland also decreased rapidly, and most of it converted to more ecological land such as garden land and water body. Under the farmland protection scenario, the tidal wetland decreased the fastest, followed by urban and rural construction land. Some construction land was projected to be converted to farmland, so that farmland would be effectively protected.
